# A Probe into the Intervention Mechanism of Yiqi Huayu Jiedu Decoction on TLR4/NLRP3 Signal Pathway in Lipopolysaccharide-Induced Acute Respiratory Distress Syndrome (ARDS) Rats

**DOI:** 10.1155/2022/3051797

**Published:** 2022-02-18

**Authors:** Yuanhong Ma, Yifan Chen, Yan Li, Yan Liu, Yurong Kong, Qiao Zou, Zhengguang Guo, Xin Li, Yan Chu, Qian Wang

**Affiliations:** ^1^Beijing University of Chinese Medicine, Beijing 100029, China; ^2^Third Affiliated Hospital of Beijing University of Chinese Medicine, Beijing 10029, China; ^3^Dongzhimen Hospital of Beijing University of Chinese Medicine, Beijing 100700, China; ^4^Institute of Basic Medical Sciences, Academy of Medical Science, Peking Union Medical College, Beijing 100005, China; ^5^Shuangqiao Hospital of Beijing, Chaoyang, Beijing 100024, China; ^6^College of Traditional Chinese Medicine, Beijing University of Chinese Medicine, Beijing 100105, China

## Abstract

**Background:**

This study discusses the anti-inflammatory mechanism of Yiqi Huayu Jiedu decoction (YQHYJD) and studies the intervening effect of YQHYJD on the inflammatory cytokines in acute respiratory distress syndrome (ARDS) rats by inhibiting the TLR4/NLRP3 signal pathway. The aim of the probe is to provide evidence to support the identification of therapeutic targets in Chinese medicine treatment, which broadens the alternatives for the treatment of ARDS.

**Method:**

A lipopolysaccharide (LPS)-induced ARDS model group is established on rats by tail vein injection. A medicine group is established on ARDS rats by prophylactic administration using YQHYJD. Materials are collected, and tests are conducted according to experimental processes.

**Result:**

The rats in the medicine group gained weight compared with those in the ARDS model group. Pathological sections from the medicine group indicated improved condition in terms of pulmonary and interstitial edema in the lung tissues of rats compared with that from the ARDS model group. The percentage of neutrophil of the medicine group was significantly brought down compared with that of the ARDS model group (*P* < 0.001). Enzyme-linked immunosorbent assay (ELISA) was used to detect the changes in the level of inflammatory cytokines. It was observed that the levels of IL-1*β* and IL-18 in serum of the medicine group significantly decreased (*P* < 0.001 and *P* < 0.01), the contents of TLR4 and NLRP3 in bronchoalveolar lavage fluid (BALF) of the medicine group decreased, and the contents of TLR4 and NLRP3 in lung tissue homogenate of the medicine group significantly decreased (*P* < 0.05, *P* < 0.001, *P* < 0.01, and *P* < 0.05). In further mass spectrum identification of the proteins from the same animal groups, it was observed that the expressions of inflammatory proteins TNFRSF1, LBP, and NOS2 of the medicine group were reduced. The differences were statistically significant.

**Conclusions:**

The pharmacological action of YQHYJD's anti-inflammatory mechanism is closely associated with the regulation of inflammatory cytokines TLR4, NLRP3, IL-1*β*, IL-18, TNFRSF1, LBP, and NOS2 on the TLR4/NLRP3 signal pathway.

## 1. Introduction

Acute respiratory distress syndrome (ARDS) is an acute respiratory disease that induces hypoxemia and pulmonary edema due to increased permeability of alveolar capillaries. The characteristics of ARDS include blurred shadows on both lungs caused by noncardiogenic pulmonary edema accompanied by severe hypoxemia [1]. ARDS is most often induced by pneumonia, septicemia, and aspiration of gastric content or severe trauma. About 10% of ICU patients worldwide suffer from ARDS [2]. In-hospital mortality on a 90-day account of moderate to serious ARDS patients stays at 43% [3]. The COVID-19 pandemic in 2019 was extremely contagious, and patients with critical conditions would develop into ARDS followed by death, making it a pressing challenge in the research of respiratory diseases to discover effective treatment approaches against ARDS. Major Western medicine therapies against ARDS include the treatment of primary disease, oxygen therapy, mechanical ventilation, extracorporeal membrane oxygenation (ECMO), drug therapy, and the use of the neuromuscular blocking agent. Symptomatic treatment remains lacking in effective approaches. The clinical drug therapy includes the use of glucocorticoid, activated protein C, antiendotoxin antibody, interferon, and pulmonary surfactant. It is usually difficult for a single symptomatic preparation to deliver ideal clinical results [1]. Chinese medicine therapy emphasizes a holistic view and syndrome differentiation. The pathogenesis of ARDS is characterized by deficiency, toxicity, and stasis. Accordingly, the treatment principle of increasing the patient's own power of healing to resist and dispel pathogenic factors is established to supplement Qi, detoxification, and break stasis, with the aim to alleviate the injury of pulmonary interstitium [4]. Meta-analysis indicates [5] that an integrated Chinese traditional and Western medicine therapy presents a promising advantage in bringing down mortality. The adoption of Chinese medicine compound combined with differential treatment demonstrates greater clinical advantages than the use of single blockers, providing a brighter outlook for the prevention and treatment of ARDS.

Yiqi Huayu Jiedu decoction [6] is a compound formula that originated from celebrated Chinese medicine doctor Huaitang Du. Previous research discovered that the herbal compound could alleviate and provide a protective effect on lung tissue injury of LPS-induced ARDS rats, reduce exudation of inflammatory cells, inhibit key proinflammatory cytokine interleukin-1*β* (IL-1*β*), and could lower protein expression of nuclear transcription factor -*κ*B (NF-*κ*B) p50, p65, and mitogen-activated protein kinase p38 (p38 MAPK) on the inflammatory pathway [7]. IL-1*β* is a key effector secreted by activated macrophages [8]. The imbalance of inflammatory cytokines will induce inflammatory reaction [9]. TLR family receptors play an important role in pathogen recognition and immune response [10]. The binding of LPS and TLR receptor activates NF-*κ*B dimer and induces acute lung injury [11–13]. Therefore, NF-*κ*B plays a critical part in immune and inflammation regulation, as it not only facilitates cytokine transcription but also remains important in the activation of NLRP3 [14–16] inflammasome. The inflammasome is a cytoplasmic complex composed of sensor molecule NOD-like receptor (NLR), adapter protein ASC, and effector protein caspase-1. NLRP3 inflammasome can be activated by multiple stimuli [17]. The component combination of the NLRP3 complex facilitates pro-caspase-1 to invert to caspase-1, followed by the activation of proinflammatory cytokines including IL-1*β* and IL-18 [18, 19]. Two steps are taken to activate the inflammasome: the inflammasome is activated and transcripted to inflammasome complexes, and then, cytokines pro-IL-1*β* and pro-IL-18 [20] are activated and released. The activation of NLRP3 can be realized in two ways. One approach involves the activation of TLR4/NF-*κ*B pathway and the expression of NLRP3, pro-IL-1*β*, and pro-IL-18. NLRP3 expression remains at a low level under normal circumstances, but when the NF-*κ*B pathway is activated, a large number of NLRP3s are transcripted and translated [21, 22]. The other approach involves pathogens in the cytoplasm and damage-associated molecular patterns (DAMPs) including NOS2 that transduce and activate NLRP3 and facilitate the assembly of inflammasomes. The activation of NLRP3 inflammasome stimulates caspase-1 to invert to an active form. Under the influence of caspase-1, precursor forms of IL-1*β* and IL-18 invert to their mature forms, causing an inflammatory response in immune reaction [23].

**Figure 1:**
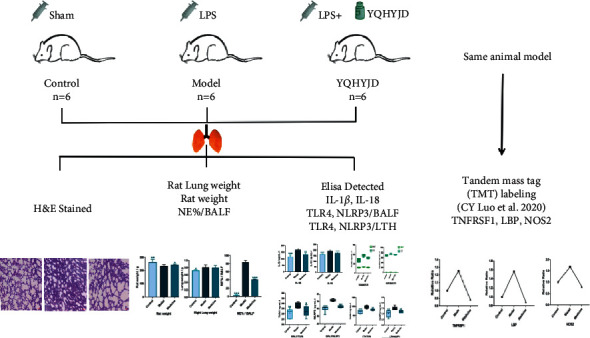
Experimental procedure.

This study further studies the anti-inflammatory activity and the functioning mechanism of the Yiqi Huayu Jiedu decoction in light of NLRP3 inflammasome-mediated pyroptosis and TLR4/NLRP3 inflammatory signaling pathway. The intervention mechanism of Yiqi Huayu Jiedu herbal compound against ARDS through multiple pathways and targets is discussed. In the meantime, proteomic mass spectrometry analysis on the same animal model indicated that Yiqi Huayu Jiedu decoction (YQHYJD) can regulate the upstream proteins including the members of tumor necrosis factor receptor superfamily (TNFRSF1), LPS-binding protein (LBP), and downstream protein NOS2 (Figure 1 Experimental Procedure).

## 2. Material and Methods

### 2.1. Experimental Animals

In total, 18 clean-grade 8-week-old male Sprague Dawley rats with an average weight of 180 ± 10 g were provided by Beijing Hfk Bioscience Co., Ltd. (license key: SCXK (Beijing) 2019-008). Experimental animals were raised at the Experimental Animal Center Barrier Laboratory of Dongzhimen Hospital of Beijing University of Chinese Medicine. The laboratory temperature was set between 22°C and 24°C, and humidity at 50%–70%. The rats were given ordinary pellet feeds with the same standard by cage feeding. The animal experiment and research were approved by the Experimental Animal Welfare and Ethics Committee of Dongzhimen Hospital of Beijing University of Chinese Medicine, no. 19–54.

### 2.2. Reagents and Instruments

LPS was purchased from Sigma, USA (batch no. L6511). TLR4 kits (96T), NLRP3 kits (96T), IL-1*β* ELISA kits, and IL-18 ELISA kits were purchased from Jiangsu Meibiao Biotechnology Co., Ltd. with batch nos. MB-1762A, MB-6880A, MB-1588B, and MB-1735B. BH2 biomicroscope was purchased from Olympus, Japan. A microplate reader was purchased from Thermo, USA. The ultraviolet spectrophotometer was purchased from Beckman Instruments, USA.

### 2.3. Chinese Medicine Required for the Experiment

Yiqi Huayu Jiedu decoction (YQHYJD) consists of eight Chinese herbal medicines [6, 24]: Astragalus membranaceus (Huang-qi) 30 g, Panax pseudoginseng (San-qi) 10 g, Paeoniae radix rubra (Chi-shao) 15 g, Fructus immaturus (Zhi-shi) 12 g, Lonicerae flos (Jin-yin-hua) 15 g, Scutellaria baicalensis (Huang-qin) 10 g, Cortex Mori (Sang-bai-pi) 15 g, and Semen Lepidii (Ting-li-zi) 15 g. The drugs used in this study were from Bozhou Yonggang Soup Tablets Factory Co., Ltd. The formulation was a traditional Chinese medicine extract. The traditional Chinese medicine was decocted twice, with water 10 times the mass of the medicine for 30 minutes each time. The two decoctions were combined, and it was concentrated by centrifugation to 1.22 g crude drug/ml and then stored at 4°C before use. The formulation was produced and extracted by the Institute of Chinese Materia Medica, China Academy of Chinese Medical Sciences (batch number:20191030). The quality control of all medicine was validated according to Chinese Pharmacopoeia and was analyzed through liquid chromatography-mass spectrometry (LC-MS) to represent the stability of the pharmaceutical ingredients of the YQHYJD compound [6].

### 2.4. Animal Grouping and Sampling

A total of 18 Sprague Dawley rats were weighed, marked, and divided according to a random number table into 3 groups, namely, control group, model group, and medicine group with 6 rats in each group. The rats were weighed and logged on daily basis. The experiment began after 1 week of adaptive feeding. The control group and model group were given distilled water by oral gavage with a dosage of 1 ml/100 g per day, once a day, for 7 continuous days. The medicine group was given Chinese medicine extract prepared using distilled water with a dosage of 12.2 g/kg, once a day, for 7 continuous days by oral gavage. Chinese medicine extract solution and distilled water were stored in the fridge at 4°C and heated before oral gavage in the water bath. Feeding ceased after oral gavage for the 7th day. Six hours after the feeding, the model group and medicine group were injected LPS solution at the dosage of 2 mg/kg by weight via tail vein, and the control group was given physiological saline at the dosage of 2 mg/kg by weight. All three groups were sampled in complete narcosis 16 hours after the injection. Intraperitoneal injection of pentobarbital sodium was administrated at the dosage of 30 mg/kg by weight. About 4-5 ml abdominal aortic blood was taken from the SD rats. Bronchoalveolar lavage fluid (BALF) was extracted from the left lung. Hematoxylin and eosin (H&E) staining was performed at the inferior lobe of the right lung. Lung tissue homogenate was prepared using the superior lobe of the right lung. BALF was processed with a low-temperature centrifuge at 4°C at 2,000 *r*/min for 5 minutes, and its supernate was preserved at −80°C for further steps of the experiment. The ARDS animal modeling was established by one single-vein injection of LPS to induce a systemic inflammatory response, which is the most common cause of ARDS. The phenotype of the animal modeling is consistent with pathologic features of the ARDS lung tissue, indicating that the modeling method is reliable [6].

### 2.5. Taking Abdominal Aortic Blood Serum and Measuring Lung Wet Weight

About 4-5 ml of abdominal aortic blood was taken and transferred into a heat-free, endotoxin-free test tube, avoiding any cell irritation. The blood was then processed by a low-temperature centrifuge at 4°C at the rate of 3,000 r/min for 30 minutes to separate the supernate and red blood cells. The blood serum was preserved at −80°C for further experiments. The tissue of the superior lobe of the right lung was dip-dried using filter paper, contained in dry and sterile tubes, and precisely weighted to measure wet weight. The lung wet weight of the three groups was compared.

### 2.6. Calculating Total Cell Number and Neutrophil Percentage in BALF

Cell sediments from centrifuged BALF were soluted with 0.1 ml physiological saline, suspended, smeared, dried, dyed, blended, washed, dried, and microscopically examined. The color of the dyed cytoplasm and nucleus could be clearly noticed. The color of dyed nucleus looked purplish red showing different levels of brightness, while the dyed cytoplasm showed light red. Particles in the cytoplasm could be clearly identified. Neutrophil count in every 20 cells on the slides was observed under an x 40 light microscope.

### 2.7. Preparation of Lung Tissue Homogenate

The lung tissue mass was rinsed in iced saline, removed of tissue blood, dried with filter paper, precisely weighed, and transferred into a homogenate tube. Homogenate media is added 9 times the volume of lung tissue mass (physiological saline). The lung tissue mass was quickly cut using ophthalmic scissors in the refrigerated aqueous bath. In order to prepare 10% lung tissue homogenate, the homogenate tube was placed in the refrigerated aqueous bath, and it was manually shaken for 30 seconds. The shaking was repeated for 6–8 times at a 30-second interval. The homogenate was then centrifuged at low temperature and low speed at 2,500 r/min for 15 minutes. The supernate was taken and transferred to a tube and was preserved at −80°C for later use.

### 2.8. Pathological Observation of Lung Tissue

The inferior lobe of the right lung after HE staining was observed under a light microscope to identify morphological changes including the changes in the alveolar septum, degree of inflammatory cell infiltration, and congestive edema of pulmonary capillary. By preparing tissue slices, dewaxing, HE staining, color separation, dehydration, and film sealing, pathologic conditions of lung tissues from each group were observed under a microscope. Lung tissue pathologic slices and HE staining were prepared and provided by the Department of Pathology of Chinese Medicine School of Beijing University of Chinese Medicine.

### 2.9. Detecting Inflammatory Cytokines Using ELISA

ELISA was performed on the previously collected abdominal aortary blood serum, BALF supernate, and lung tissue homogenate according to instructions. Protein antibody-coated microbody was added into specimen, standard sample, and detection antibody was labeled and then hatched and thoroughly rinsed. The absorbance was calculated using a microplate reader at 450 nm to confirm the sample concentration. IL-1*β* and IL-18 were detected in the blood serum, and TLR4 and NLRP3 were detected in BALF and lung tissue homogenate, respectively.

### 2.10. Protein Quantitative Analysis Using Tandem Mass Tag (TMT)

The modeling and grouping methods of SD rats followed the same instructions as in bulletin 2.4. Samples of the left lung tissue were taken for proteomic screening, followed by splitting decomposition, centrifugation, protein extraction, detection of protein concentration, proteolysis, and peptide purification and quantification. The qualitative and quantitative analysis of protein was conducted using liquid chromatography-mass spectrometry (LCMS). The detailed procedure was listed in reference item 6. Protein data were retrieved from the database of the Proteome Discoverer software suite (Version 2.1, Thermo Fisher Scientific). The total protein (TP) was retrieved in 3 sample groups, and the relative amount of protein was determined in each group. Proteins with *P* value <0.05 and absolute value of fold change (FC)>0.1 were considered to be differential expression proteins (DEPs). Contrast detection analysis on inflammatory proteins TNFRSF1, LBP, and NOS2 was conducted.

### 2.11. Statistical Processing

A database with all research indexes was set up, and the statistics was analyzed applying SPSS 21.0, with measurement data represented by *X* ± *S*. Difference comparison between each group was analyzed with one-way ANOVA, and multiple comparisons were detected using LSD. *P* value <0.05 was considered to be of statistical significance. Software GraphPad Prism 7 was applied for charting.

## 3. Experimental Results

### 3.1. ARDS Rats Conditions

The experimental animal model was an acute lung injury model related to LPS-induced pyemia. The time duration of the experimental process was relatively short. The control group demonstrated insignificant changes during the experiment with a good mental state, agility and freedom in movement, and bright and soft-smooth hair. The model group started to show deteriorating mental conditions, reduced movement, slow in reaction, sleep-prone languor, withered and yellow hair lacking in luster, cyanosis in lips and claws, and unformed stool. The medicine group demonstrated similar but relatively lighter conditions compared to the model group.

### 3.2. Pathological Observation of Lung Tissue

**Figure 2:**
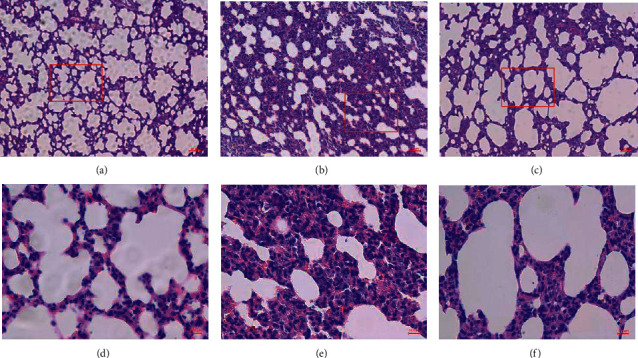
Pathological sections of lung tissues of experiment groups. (a) Control group ×100. (b) Model group ×100. (c) Medicine group ×100. (d) Control group ×400. (e) Model group ×400. (f) Medicine group ×400.

In the lung tissue of the control group, clear and complete alveolar structure without congestive edema of the alveolar wall could be observed, and alveolar cavity structure was clear to be noticed (Figures 2(a) and 2(d)). In the lung tissue of the model group, unclear alveolar was observed with obvious damage in structure, congestive edema in pulmonary interstitium, thickened alveolar wall, and shrunk alveolar cavity with a large amount of inflammatory exudates and red cells. A large amount of secretions in the bronchus and congestive edema of blood capillaries were also observed (Figures 2(b) and 2(e)). Pathological characteristics of lung tissue in the medicine group showed improved conditions in terms of alveolar wall edema and pulmonary interstitium edema (Figures 2(c) and 2(f)).

### 3.3. Changes in Animal Body Weight, Lung Wet Weight, and Neutrophil in BALF

#### 3.3.1. Animal Body Weight

**Figure 3:**
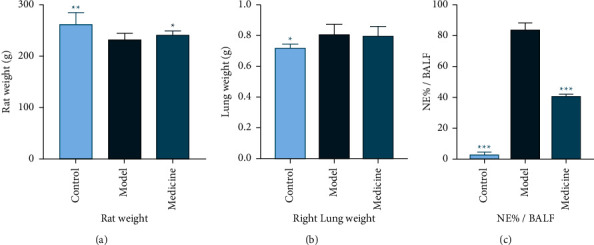
Changes in rats' body weight, lung wet weight, and neutrophils in BALF. (a) Body weight in 3 experimental groups. (b) Right lung wet weight in 3 experimental groups. (c) Neutrophil percentage in 3 experimental groups. Note: in comparison with model group, ^*∗*^*p* < 0.05, ^*∗∗*^*p* < 0.01, and ^*∗∗∗*^*p* < 0.001.

Compared with the control group, the model group ARDS rats lost weight (*P* < 0.01). Compared with the model group, the medicine group rats gained weight (*P* < 0.05). This indicates that YQHYJD herbal compound has the effect to improve the overall physical function of ARDS rats, and no adverse effect was observed (Table 1, Figure 3(a)).

#### 3.3.2. Wet Weight of Right Lung Tissue

Compared with the control group, the model group most significantly gained the lung wet weight (*P* < 0.05), indicating that the model group suffered from the severest pulmonary edema. The lung wet weight of the medicine group showed no obvious change compared to that of the model group (Table 1 and Figure 3(b)).

#### 3.3.3. Neutrophil Percentage in BALF

Neutrophil percentage in the model group increased (*P* < 0.001) compared with the control group, while the neutrophil percentage in the medicine group significantly decreased (*P* < 0.001) compared with the model group (Table 1 and Figure 3(c)).

### 3.4. IL-1*β* and IL-18 Expressions in Blood Serum, and TLR4 and NLRP3 Expressions in BALF and Lung Tissue Homogenate

#### 3.4.1. IL-1*β* and IL-18 in Blood Serum

**Figure 4:**
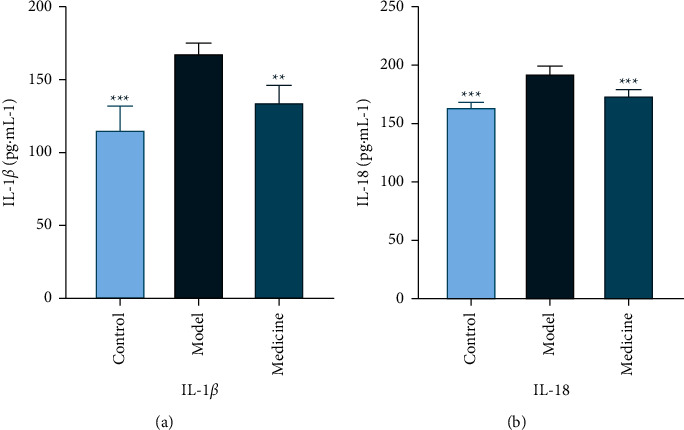
Comparison of expression levels of IL-1*β* and IL-18 in blood serum. (a) IL-1*β* content in blood serum. (b) IL-18 content in blood serum. Note: in comparison with model group, ^*∗*^*p* < 0.05, ^*∗∗*^*p* < 0.01, and ^*∗∗∗*^*p* < 0.001.

IL-1*β* expression raised in the model group blood serum compared with control group (*P* < 0.05). IL-1*β* expression in the medicine group blood serum significantly lowered (*P* < 0.001) compared with the model group. The IL-1*β* content is measured by pg/ml (Table 2 and Figure 4(a)). IL-18 expression raised in the model group blood serum compared with the control group (*P* < 0.001). IL-18 expression in the medicine group blood serum significantly lowered (*P* < 0.01) compared with the model group. The IL-18 content is measured by pg/ml (Table 2 and Figure 4(b)).

#### 3.4.2. TLR4 and NLRP3 in BALF

**Figure 5:**
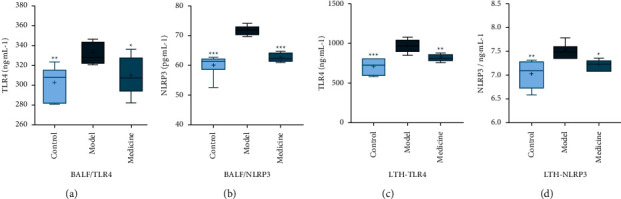
Comparison of expression levels of TLR4 and NLRP3 in BALF and lung tissue homogenate. (a) TLR4 content in BALF. (b) NLRP3 content in BALF. (c) TLR4 content in lung tissue homogenate. (d) NLRP3 content in lung tissue homogenate. Note: in comparison with model group, ^*∗*^*p* < 0.05, ^*∗∗*^*p* < 0.01, and ^*∗∗∗*^*p* < 0.001.

TLR4 expression in BALF of the model group increased (*P* < 0.01) compared with the control group. TLR4 expression in BALF of the medicine group significantly decreased (*P* < 0.05) compared with the model group. TLR4 content in BALF from 3 groups was measured by pg/ml(Table 3 and Figure 5(a)). Expression level of NLRP3 in BALF of the model group increased (*P* < 0.001) compared with the control group. NLRP3 expression in BALF of the medicine group significantly decreased (*P* < 0.001) compared with the model group. NLRP3 content in BALF from 3 groups was measured by pg/ml (Table 3 and Figure 5(b)).

#### 3.4.3. TLR4 and NLRP3 in Lung Tissue Homogenate

TLR4 level in lung tissue homogenate of the model group raised (*P* < 0.001) compared with the control group. TLR4 level significantly lowered in lung tissue homogenate of the medicine group compared with the model group (*P* < 0.01). TLR4 content (pg/ml) is shown in lung tissue homogenate of 3 groups in Table 4 and Figure 5(c). Expression level of NLRP3 in lung tissue homogenate of the model group increased (*P* < 0.01) compared with the control group. NLRP3 level in lung tissue homogenate of the medicine group significantly decreased (*P* < 0.05) compared with the model group. NLRP3 content in lung tissue homogenate of 3 groups is shown in Table 4 and Figure 5(d).

### 3.5. Comparison of TLR4 and NLRP3 Content Levels in BALF and Lung Tissue Homogenate

**Figure 6:**
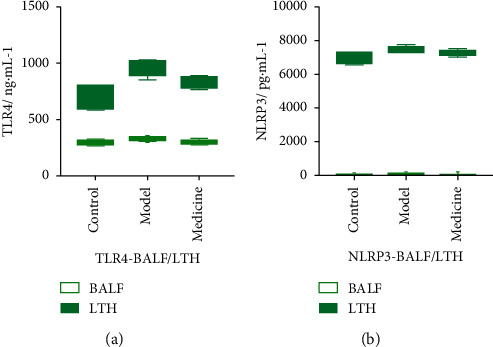
Comparison of TLR4 and NLRP3 contents in BALF and lung tissue homogenate. (a) Comparison of TLR4 in BALF/lung tissue homogenate in 3 experimental groups. (b) Comparison of NLRP3 in BALF/lung tissue homogenate in 3 experimental groups.

The content level of TLR4 in lung tissue homogenate was higher than that in BALF (*P* < 0.001), indicating that lung tissue homogenate contained a larger amount of TLR4 and that LPS-induced ARDS modeling enhanced the expression of TLR4 receptor (Figure 6(a)). The content level of NLRP3 in lung tissue homogenate was higher than that in BALF (*P* < 0.001), indicating that NLRP3 increased in lung tissue homogenate and that LPS-induced ARDS modeling facilitated the activation of NLRP3 and triggered inflammatory response (Figure 6(b)).

### 3.6. Proteomic Mass Spectrometry Analysis

**Figure 7:**
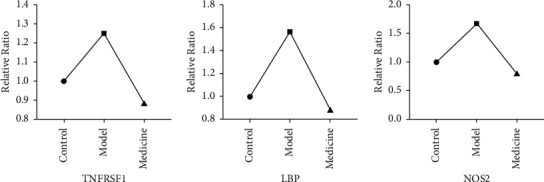
Changes in differential protein using proteomic mass spectrometry analysis.

Proteomic mass spectrometry analysis was applied to further verify that YQHYJD could regulate the upstream proteins TNFRSF1 and LBP and downstream protein NOS2 on the TLR4/NLRP3 pathway. The research team also conducted a preliminary mass spectrographic analysis on the accessory lobe of the right lung of ARDS rats. Combining two analyses and experiment results, it was found out that upstream proteins TNFRSF1 and LBP and downstream protein NOS2 on the TLR4/NLRP3 pathway were upregulated in the model group compared with the control group, while upstream proteins TNFRSF1 and LBP and downstream protein NOS2 on the same pathway were downregulated in the medicine group compared with model group. This further proved the regulating effect of the YQHYJD compound on major proteins on the particular pathway (differential protein content is shown in Table 5 and Figure 7).

## 4. Discussion

ARDS is an inflammatory response caused by increased permeability of alveolar epithelial tissue. This inflammatory response is induced by the activation of an inflammatory signaling pathway that triggers the release of inflammatory cytokines. Pathological sections of lung tissues indicated that the alveolar structure of the model group exhibited obvious injury with congestive edema in pulmonary interstitium and a large amount of inflammatory exudates in the alveolar cavity. Under prophylactic administration of YQHYJD, the medicine group demonstrated improved conditions in terms of alveolar injury and pulmonary interstitium edema. This was consistent with pathological results of inflammatory response and confirmed the general anti-inflammatory effect of YQHYJD. It was observed from the inflammatory exudates of the model group that lung wet weight and neutrophil content in BALF increased, while the inflammatory response of the medicine group was regulated demonstrating reduced pulmonary injury. TLR4/NLRP3 pathway is a typical inflammatory pathway. Its activation stimulates the release of inflammatory cytokines IL-1*β* and IL-18, which further aggravates the inflammatory response. Preliminary research studies indicated that YQHYJD could regulate the expressions of major proteins NF-*κ*B p50, p65, and p38 MAPK in the inflammatory pathway [7]. Experimental results of the medicine group showed that content levels of IL-1*β* and IL-18 in blood serum on the TLR4/NLRP3 pathway decreased and the expressions of inflammatory proteins TLR4 and NLRP3 in BALF and lung tissue homogenate were inhibited, compared with the model group. This was closely correlated with YQHYJD's effect of inhibiting TLR4 activation, NF-*κ*B transcription, and the assembly of NLRP3 inflammasomes that helped to reduce the IL-1*β* and IL-18 release. To further verify the relevance between YQHYJD's pharmacological actions and TLR4/NLRP3 pathway, proteomic mass spectrometry analysis was conducted to detect the premedicinal and postmedicinal changes in upstream proteins TNFRSF1 and LBP and downstream protein NOS2. The analysis indicated YQHYJD could inhibit not only the expression of upstream proteins TNFRSF1 and LBP and downstream protein NOS2 on the pathway but also the activation of NLRP3 inflammasomes, bring down the content level of inflammatory protein, curb the binding of TLR4 receptor, and downregulate the inflammatory response. The general anti-inflammatory mechanism of YQHYJD was further proved.

**Figure 8:**
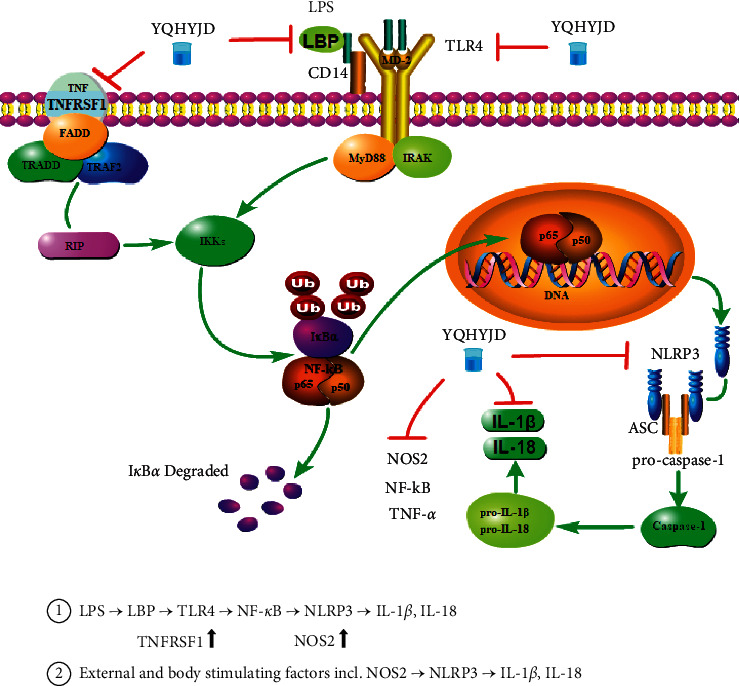
TLR4/NLRP3 signal pathway.

Inflammasomes in inflammatory cells and immunocytes play an important role in immune reaction. NLRP3 inflammasome is being widely studied. The inflammatory cell is stimulated into an active form, NLRP3 inflammasome functions together with pro-IL-1*β* and ligand ASC [25]. NLRP3 inflammasome can be activated under 2 conditions: first, signal activation and, second, activation of caspase-1. When externally irritated, TLR4 binds with LPS and triggers the activation of NF-kB on the signal pathway, by upregulating NLRP3 and pro-IL-1*β*. Activated NLRP3 assembles with pro-IL-1*β* and ASC into NLRP3 inflammasome, which activates pro-caspase-1 into cleaved caspase-1. By enzymatic action, pro-IL-1*β* and pro-IL-18 are released in mature forms causing inflammatory response [26–29] (Figure 8 signal pathway).

YQHYJD is composed of *Astragalus* membranaceus (Huang-qi), Panax pseudoginseng (San-qi), Paeoniae radix rubra (Chi-shao), Fructus immaturus (Zhi-shi), Lonicerae flos (Jin-yin-hua), Scutellaria baicalensis (Huang-qin), Cortex Mori (Sang-bai-pi), and Semen lepidii (Ting-li-zi). Research studies revealed that the major active ingredient astragaloside IV (AS-IV) derived from *Astragalus* membranaceus could significantly reduce the expression of TLR4 and downstream adaptor proteins, regulate NF-*κ*B phosphorylation, and inhibit the activation of NLRP3 inflammasome [30]. The major active ingredient Panax notoginseng saponins (PNS) from Panax pseudoginseng has the pharmacological effect of anti-inflammation and antioxidation, which plays a part in regulating mitochondrial energy metabolism, oxidative stress, apoptosis, mitochondrial autophagy, and membrane channel status [31]. Paeoniae radix rubra is characterized by its effect of anti-inflammation and immunoregulation. It also inhibits the activation of NF-*κ*B pathway and downregulates the protein expression of MAPK [32]. Fructus immaturus extract is an ingredient with anti-inflammatory activity, which is effective in inhibiting MAPK and NF-*κ*B signal pathway activation [33]. Lonicerae flos is effective in anti-inflammation, antibacteria, and antivirus. Lonicerae flos extract HS-23 can reduce septicemia mortality, enhance bacterial clearance rate, and relieve multiple organ failures. Lonicerae flos's action mechanism is related to reducing the protein expression of TLR4 [34]. Lonicerae flos extract flavonoids are effective in regulating inflammatory cytokines and mediators, which are related to the regulating of nuclear translocation of NF-*κ*B and phosphorylated Akt [35]. Lonicerae flos extract luteolin can significantly inhibit NF-*κ*B activation and plays a role in anti-inflammation by lowering Ca^2+^ level to regulate expression of TNF-*α*, IL-6, IL-8, GM-CSF, and COX-2 [36]. Scutellaria baicalensis extract baicalin (BAI) can regulate inflammatory response by inhibiting the activation of NLRP3 inflammasome and TLR4/NF-*κ*B signal pathway [37]. Scutellaria baicalensis extract baicalin can reduce inflammatory response by downregulating the activation of NF-*κ*B and p38 MAPK and by reducing TLR4 and MyD88 expressions [38]. Cortex Mori extracts mulberroside A and oxyresveratrol are effective in anti-inflammation and antioxidation, which might be related to the regulation of iNOS expression by downregulating the binding activity of NF-*κ*B and COX-2 activities [39]. Semen lepidii extract total flavonoids (TFH) can regulate the nuclear translocation of activated NF-*κ*B, downregulate TLR4 and NF-*κ*B, and are effective in antifibrosis [40].

Compared with ARDS treatment using a single blocker, this study studied the integrated regulating effect of YQHYJD on the TLR4/NLRP3 pathway in ARDS treatment. The micromechanism of traditional Chinese medicine compound was thoroughly explored. This innovative research provided evidence for the identification of therapeutic targets in ARDS treatment using Chinese medicine therapy, introducing a novel approach for the treatment of ARDS. However, Chinese medicine compound is composed of complex ingredients. This study did not focus on the action mechanism of any specified ingredient from the compound against ARDS, nor was it verified by cellular experiments. This provided a perspective for future research.

## 5. Conclusions

Research indicated that by prophylactic administration of YQHYJD, lung tissue injury of LPS-induced ARDS rats was alleviated, the levels of inflammatory cytokines IL-1*β* and IL-18 in blood serum were downregulated after the activation of the TLR4/NLRP3 pathway, the content levels of inflammatory proteins TLR4 and NLRP3 in BALF and lung tissue homogenate were reduced. Pre- and post-YQHYJD treatments were further detected to observe the changes in upstream proteins TNFRSF1 and LBP and downstream protein NOS2 on the TLR4/NLRP3 pathway. It was observed that the Chinese medicine compound could inhibit the expression of upstream proteins TNFRSF1 and LBP and downstream protein NOS2, confirming YQHYJD's anti-inflammatory effect against ARDS through TLR4/NLRP3 pathway.

## Figures and Tables

**Table 1:** Body weight, right lung wet weight, and neutrophil percentage in BALF in 3 experimental groups.

Group	*n*	Body weight (g)	Right lung wet weight (g)	Neutrophil percentage (%)
Control	6	262.00 ± 23.580^*∗∗*^	0.720 ± 0.028^*∗*^	2.67 ± 1.967^*∗∗∗*^
Model	6	232.50 ± 13.722	0.805 ± 0.074	83.67 ± 5.007
Medicine	6	241.17 ± 8.954^*∗*^	0.800 ± 0.065	40.33 ± 2.066^*∗∗∗*^
*P* value	*P*=0.021	*P*=0.046	*P* < 0.001

Note: in comparison with model group, ^*∗*^*p* < 0.05, ^*∗∗*^*p* < 0.01, and ^*∗∗∗*^*p* < 0.001.

**Table 2:** IL-1*β* and IL-18 expressions in blood serum of 3 experimental groups.

Group	*n*	IL-1*β* (pg·mL^−1^)	IL-18 (pg·mL^−1^)
Control	6	114.428 ± 18.143^*∗∗∗*^	163.189 ± 5.721^*∗∗∗*^
Model	6	167.950 ± 7.577	191.427 ± 7.955
Medicine	6	133.495 ± 12.963^*∗∗*^	172.699 ± 6.942^*∗∗∗*^
*P* value	*P*=0.001	*P* < 0.01

Note: in comparison with model group, ^*∗*^*p* < 0.05, ^*∗∗*^*p* < 0.01, and ^*∗∗∗*^*p* < 0.001.

**Table 3:** TLR4 and NLRP3 contents in BALF of 3 experimental groups.

Group	*n*	TLR4 (ng·mL^−1^)	NLRP3 (pg·mL^−1^)
Control	6	331.439 ± 10.505^*∗∗*^	62.122 ± 3.758^*∗∗∗*^
Model	6	302.613 ± 17.218	72.267 ± 2.109
Medicine	6	309.458 ± 19.090^*∗*^	62.465 ± 1.115^*∗∗∗*^
*P* value	*P*=0.018	*P* < 0.001

Note: in comparison with model group, ^*∗*^*p* < 0.05, ^*∗∗*^*p* < 0.01, and ^*∗∗∗*^*p* < 0.001.

**Table 4:** TLR4 and NLRP3 contents in lung tissue homogenate of 3 experimental groups.

Group	*n*	TLR4 (ng·mL^−1^)	NLRP3 (ng·mL^−1^)
Control	6	710.132 ± 93.235^*∗∗∗*^	7.025 ± 0.295^*∗∗*^
Model	6	971.021 ± 84.300	7.512 ± 0.158
Medicine	6	819.383 ± 44.624^*∗∗*^	7.214 ± 0.112^*∗*^
*P* value	*P* < 0.001	*P*=0.003

Note: in comparison with model group, ^*∗*^*p* < 0.05, ^*∗∗*^*p* < 0.01, and ^*∗∗∗*^*p* < 0.001.

**Table 5:** Differential protein content by proteomic mass spectrometry analysis.

Protein name	Control	Model	Medicine	Model/control	Medicine/model
TNFRSF1	106.90	133.7	118.40	1.251	0.886
LBP	86.60	135.15	119.70	1.561	0.886
NOS2	82.55	138.65	111.60	1.680	0.805

Note: Three types of inflammatory proteins were composite samples of lung tissues from each group, no UOM, 100 as base value, and others reflect relative ratio.

## Data Availability

The original data in this study are included in this article, and the authors have affirmed the accuracy of the data.
